# Analytical model of the feto-placental vascular system: consideration of placental oxygen transport

**DOI:** 10.1098/rsos.180219

**Published:** 2018-04-11

**Authors:** Parisa Mirbod

**Affiliations:** Department of Mechanical and Aeronautical Engineering, Clarkson University, Potsdam, NY, USA

**Keywords:** theoretical modelling, human and mouse placental blood flow, feto-placental circulation, mass transport, convective and diffusive resistances to oxygen transport

## Abstract

The placenta is a transient vascular organ that enables nutrients and blood gases to be exchanged between fetal and maternal circulations. Herein, the structure and oxygen diffusion across the trophoblast membrane between the fetal and maternal red blood cells in the feto-placental vasculature system in both human and mouse placentas are presented together as a functional unit. Previous models have claimed that the most efficient fetal blood flow relies upon structures containing a number of ‘conductive’ symmetrical branches, offering a path of minimal resistance that maximizes blood flow to the terminal villi, where oxygen diffusion occurs. However, most of these models have disregarded the actual descriptions of the exchange at the level of the intermediate and terminal villi. We are proposing a ‘mixed model’ whereby both ‘conductive’ and ‘terminal’ villi are presumed to be present at the end of single (in human) or multiple (in mouse) pregnancies. We predict an optimal number of 18 and 22 bifurcation levels in the human and the mouse placentas, respectively. Wherever possible, we have compared our model's predictions with experimental results reported in the literature and found close agreement between them.

## 1. Introduction

The placenta is an effective reporter of information about placental and fetal exposures and their developmental consequences [1]. Its most important function is to exchange endogenous and exogenous substances that enable an adequate supply of oxygen and nutrients and the excretion of fetal metabolic waste [2–6]. Placental development and feto-placental angiogenesis are critical for successful gestation. To date, placental research has been extremely challenging due to the *in vivo* ethical limitations and the manipulation of the *ex vivo* organ. In addition, translating animal models to human functions is problematic because of the differences in the structure and biochemistry of human and animal placentas [7]. Therefore, despite numerous studies of multiple species and the use of experimental human placental functional unit [8–15] the functional relationships and mass transfer between the maternal and fetal blood flows at the terminal villus are poorly understood. For instance, extensive studies in multiple species, as well as studies using human placental lobule have been performed to understand both the bidirectional transfer of solutes, including nutrients and xenobiotics, and the effects of flow and solute properties (early studies were performed in sheep [8]). Researchers studied ‘flow-limited transfer’ and ‘diffusional-limited transfer’ of substances in a pig due to the relative importance of the trophoblast according to molecular properties of the analytics [9]. ‘Multi-villous flow’ and its effects on transfer efficacy have been also reported [10]. A consideration was given to the putative question of whether or not hypoxic venous constriction on fetal to maternal blood flow matching affects the transplacental exchange rate [11,12], as well as the ‘compartmentalization’ of blood flow in relation to the integrative studies of amino acid transfer [13]. More recent studies have tackled the modelling of placental solute uptake from the maternal circulation by assuming the placenta to function as a porous medium [14,15]. These studies have also estimated fetal capillary acquisition of oxygen by considering pressure drops through capillaries accounting for sinusoids, luminal axis length and capillary branching angiogenesis [16].

Furthermore, clinical and anatomical studies of the placenta and its vasculature have confirmed that its efficiency is determined by the structure of the vasculature as it moves through the large vessels to the small capillary vessels, that contribute to the gas exchange, as well as the size and shape of the placenta itself [17]. Thus, an account of the details of structural analysis and mass transport in the placenta plays a critical role in understanding its function. Analytical and computational models reveal a relationship between the structure and the function of the placenta, which can, in turn, lead to a better understanding of human pregnancy. To understand how mass transfers between the fetus and mother, we must analyse the structure of the vasculature from large vessels to the small capillary vessels that contribute to gas and nutrient exchange. In this study, we are proposing a model of the feto-placental vasculature system. The model includes idealized anatomical detail from the umbilical cord through the fetal capillaries and a description of the feto-placental vasculature that accounts for the mass transfer properties of both human and mouse placentas. We primarily focus on oxygen transfer through the system.

Other researchers have recently investigated some of the individual elements of this model. For example, one study attempts to understand the influence of a morphology description on the respiratory gas exchange placenta model [17]. Other recent studies have tried to use computational modelling to understand how the feto-placental structure influences oxygen transport [18–20]. Others have used computational modelling of the structure–function relationship in human placental terminal villi [19,20] or have modelled oxygen transport in human placental terminal villi [21]. The human feto-placental vasculature and its branching structure from the point of cord insertion in the human placenta have been also analysed [22–26]. Researchers have also reported the impact of the pulsatile fetal blood flow in the vasculature system of the mouse placenta [27]. However, none of these researches evaluated the optimal structure of the feto-placental vasculature network with respect to oxygen transport, which is the focus of this study. We are demonstrating that the placenta can be efficiently modelled by replacing a vessel network composed of distinct elements and considering both convective and diffusive properties, as has been proposed for the airflow and gas exchange in the lungs [28–30].

**Figure 1.**
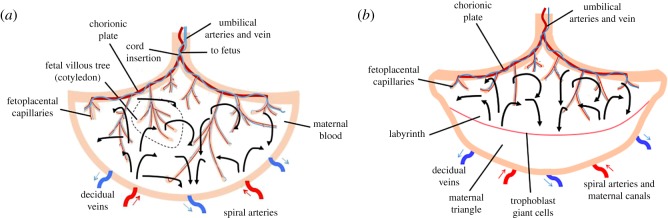
Schematic model of the feto- and materno-placental vasculature system. (*a*) Human, (*b*) mouse. Solid line arrows represent the maternal blood flow. Dashed line represents the fetal villous tree (cotyledon). Red arrows show the spiral arteries and blue arrows represent decidual veins.

In addition, in order to offer a revised model of the placenta, we are using comparisons of the morphology of human and mouse placentas to understand how oxygen transport occurs. Not only are there morphological similarities between human and mouse placentas but also the mechanisms of materno-fetal solute and gas exchange are likely similar between these two species [7]. Both placentas are discoidal in shape and are of haemochorial type; that is, they contain fetal capillaries surrounded by layers of trophoblasts directly bathing in maternal blood [31,32]. There are no maternal endothelial cells in the exchange region of the placenta in either human or mouse placentas [33,34]. Of course, distinct differences also exist between the human and mouse placenta, including: (1) size, (2) geometric arrangement of the maternal and fetal circulation system, (3) concentration and hydrostatic gradients between maternal and fetal plasma [33,34], (4) trophoblast tissue differences by virtue of which the human placenta is haemomonochorial, while the mouse placenta is haemotrichorial, (5) circulation within the placenta, by which the multi-villous arrangement and corresponding ‘open-pool’ maternal circulation in the human placenta differ distinctly from the close-circuit labyrinthine circulation of the mouse placenta [35], and (6) endothelial structural differences between mice and human. For instance, the mouse placenta has a fenestrated endothelium and only one basement membrane in the placental barrier, whereas the human has a trophoblast, as well as an endothelial basement membrane and a continuous endothelium [31,32]. Additional differences between the vascular structural organization of the human and mouse placentas have been described [33,36,37] (figure 1*a,b*).

As shown in figure 1, the vascular morphology of both human and mouse placentas includes spiral arteries supplying and draining decidual veins from the maternal side, as well as a dense network of vessels in the feto-placental tree from the fetal side. The feto-placental vessels have a tree-like structure and are located in the maternal blood basin, i.e. intervillous space [38]. Fetal arterial blood flows from the umbilical arteries to the capillaries and returns to the umbilical vein (figure 1*a*). In the human placenta, umbilical vessels branch into six to eight generations of chorionic plate arteries that feed 60–100 cotyledons [39,40]. Each cotyledon is made up of villous trees that house the feto-placental vessels. These vessels bifurcate many times before branching into elongated capillaries that bulge from the distal end of the villous trees [22,23] to create a large surface area for exchange. The mouse placenta is comparable to a single human cotyledon [41,42] (figure 1*b*). We have modelled the human and mouse placentas separately using parameters that reflect this difference.

In summary, we hypothesize that the feto-placental vascular systems, specifically the number of vascular bifurcations, are constructed such that they promote the most efficient access of fetal blood flow and oxygen transfer to the fetus. We are proposing a mathematical model for a feto-placental vasculature system in which a combination of convection and diffusion resistance provides oxygen transport in the placenta. This model represents the optimal structure of the system in both mouse and human placentas. To our knowledge, no other investigators have viewed the structure of the feto-placental vasculature network in terms of its specific function in oxygen transport while also considering the actual concentration of oxygen dissolved in blood plasma and oxygen bound to haemoglobin. After estimating the optimal number of bifurcation levels yielding the minimum total oxygen transfer resistance for mass transport between the fetal and maternal blood in the human and mouse placentas, we evaluate some specific parameters, such as optimum oxygen volume flow rate through the vasculature system and the fetal capillary diameter, that are usually difficult to measure experimentally.

### 1.1. Analytical modelling of the feto-placental vasculature system using flow resistances

**Figure 2.**
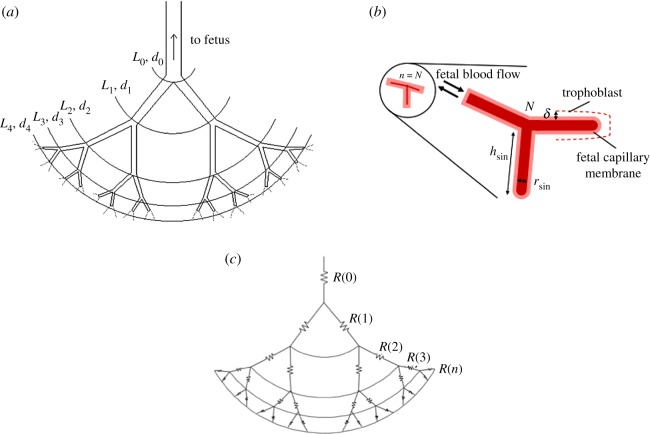
Representative analytical model. (*a*) Feto-placental vasculature system. (*b*) Fetal capillary system (note that this diagrammatic representation of villous vasculature ignores the existence of arterioles and venules and treats the villous as having a single vessel). (*c*) The system resistivity chart with respect to the oxygen transport. The fetal vessels are assumed to be cylindrical channels where the Hagen–Poiseuille equation can be applied; the fetal capillary is assumed to have a spherical shape; and the vasculature system is assumed to be symmetrical. Here, *R, L, d* and *N* represent the total resistance, the length and diameter of the vessels, and the number of bifurcation levels, respectively. The pictures are not to scale.

Figure 2 shows a general two-dimensional structure and our representative model of the feto-placental vasculature system from the umbilical artery through bifurcations to the feto-placental capillaries. To develop a mathematical model for the flow structure, we use both convective and diffusive resistance to oxygen transport by considering the vessels in the vasculature system as resistors arranged either in parallel or in series (figure 2*c*). It should be noted that in the proposed model the convective resistances have been applied through the feto-placental vasculature trees, while the diffusive flux resistances have been considered in the fetal capillaries. Thus, all convective resistances in the capillaries are neglected. A similar approach has been used by [28,29] to define the characteristics of the alveolar sacs in the lungs. In addition, there are some distinctions between our proposed model and the model reported by [29] including: (1) the method to define the diffusive flux resistance in the vasculature system, (2) the calculation, in detail, using the Hill equation and experimental parameters, of the concentration of oxygen dissolved in the fetal blood plasma and bound to haemoglobin, (3) the proposed model considers the number of cotyledons and fetal capillaries in both human and mouse placenta and (4) even though the fetal capillaries are very complicated, we assumed they are cylindrical capillaries while the alveolar sacs have been considered spherical. Wherever possible, we have also analysed and compared our theoretical results with the experimental data.

Although the placental villi from their arterial origin to the lacunae are morphologically separated into conductive, intermediate and terminal villi, in this study, for simplicity, we will take into account only conductive and terminal villi. Note that the optimal number of bifurcation levels giving rise to the easiest fetal blood flow through the vasculature system was obtained by minimizing the total convective and diffusive resistances to oxygen transport as follows.

#### 1.1.1. Convective resistance through the feto-placental vasculature system and the conductive villi

To derive the convective resistance through the network of vessels, we must first characterize the flow regime. Following other researchers [39], we considered the normal mean fetal blood flow rate in the umbilical arteries that feed the placenta during the third trimester of pregnancy to be 500 ml min^−1^, the diameter of the umbilical artery to be 4 mm [43], and the viscosity and density of blood [14] to be 4 × 10^−3^ Pa s and 10^3^ kg m^−3^, respectively (as listed in table 1). We then found that the maximum Reynolds number of the fetal blood flow in the vessels would be approximately 0.663, which is compatible with a laminar flow regime. The maximum Reynolds number at term (i.e. embryonic days (E)17.5) in the mouse placenta was approximately 4.65 given an estimated mean arterial blood flow velocity of 31 mm s^−1^ [36] and an umbilical artery diameter of 0.54 mm [45]. Note that the maximum Reynolds number for a mouse at (E)13.5 and (E)15.5 will be approximately 3.75. Thus, by considering the fetal vessels as cylindrical channels, the fetal blood flow rate for a given pressure loss Δ*P*_Nf_ of each of the two channels in the generation Nf can be given by the well-known Hagen–Poiseuille equation [46] as

1.1 $$\Delta P_{\mathrm{Nf}}=Q_{\mathrm{Nf}}\;\frac{128\mu_{b}L_{\mathrm{Nf}}}{\pi d_{\mathrm{Nf}}^{4}}.$$

**Table 1.** The typical parameter values of human and mouse placenta based on experimental measurements.

			mouse ref.		
			stages of gestation		
description	unit	human in the third trimester (references)	E13.5	E15.5	E17.5
umbilical artery length, *L*_0_	m	0.3 − 0.7 [44]	0.0056 [45]	0.0056 [45]	0.0056 [45]
umbilical artery diam. from CT, *d*_0_	m	0.004 [43]	0.00032 [36]	0.00046 [36]	0.00054 [36]
coefficient of diffusion (oxygen), *D*_ox_	m^2^ s^−1^	1.7 × 10^−9^ [14]	1.7 × 10^−9^ [14]	1.7 × 10^−9^ [14]	1.7 × 10^−9^ [14]
experimentally measured mean umbilical artery blood velocity, *V*_mean_	m s^−1^	—	2 [33]	25 [33]	31 [33]
umbilical artery blood flow rate, *Q*	ml min^−1^	500 [39]	0.12 [36]	0.246 [36]	0.426 [36]
blood viscosity, *μ*_b_	Pa s	4 × 10^−3^	4 × 10^−3^	4 × 10^−3^	4 × 10^−3^
blood density, *ρ*_b_	kg m^−3^	1000	1000	1000	1000
partial input pressure, *P*_input_	mmHg	8 − 18 [21]	—	—	—
partial output pressure, *P*_output_	mmHg	22 − 37 [21]	—	—	—
the number of cotyledons, *α*	—	60–100 [39,40]	1	1	1

Hence, the resistance of the fetal blood vessels in the placenta becomes

1.2 $$R_{\mathrm{conv}.}^{b}(\text{Nf})=\frac{\Delta P_{\mathrm{Nf}}}{Q_{\mathrm{Nf}}}=\frac{128\mu_{b}L_{\mathrm{Nf}}}{\pi d_{\mathrm{Nf}}^{4}}.$$

Here, the subscript Nf refers to the channel of the Nf^th^ bifurcation level, Nf = 0 corresponds to the umbilical artery and *L*_0_ and *d*_0_ denote the length and diameter of the umbilical artery, respectively. Δ*P*_Nf_, *Q*_Nf_, *ρ*_b_, *μ*_b_, d_Nf_ and *L*_Nf_ specify the pressure drop of the *N*^th^ bifurcation level, fetal blood volume flow rate, blood density, dynamic viscosity, channel diameter and the length of the Nf^th^ generation, respectively.

Consequently, we can show that the minimum flow resistance at a bifurcation for a fixed total volume (i.e. *L*_Nf−1_*d*_Nf−1_^2^ + *L*_Nf_*d*_Nf_^2^ = Constant) was achieved when the ratio between consecutive channel diameters followed Murray's law [47] as $d_{\mathrm{Nf}}\text{/}\;d_{\mathrm{Nf}-1}=2^{-1/3}.$ This is because the model geometry was assumed to be symmetrical and each channel was divided into two equal channels (figure 2*a*). This ratio was first reported in physiology by [48] and later by [47,49] for a fully developed laminar flow regime through the system. This relationship also reveals that the optimal $d_{\mathrm{Nf}}\text{/}d_{\mathrm{Nf}-1}$ is independent of the assumed channel length; therefore, it is independent of the geometry [28] and it holds for any bifurcation angle and any set of two channel lengths [47]. In a symmetrical geometry, we also expected the same pressure drop sequence from the umbilical artery to the feto-placental capillaries and from the feto-placental capillaries to the umbilical vein (i.e. the pressure drop at each level should be equal). This gave us the ratio between consecutive channel lengths as [28] $L_{\mathrm{Nf}}\text{/}\;L_{\mathrm{Nf}-1}=2^{-1/3}$. Using these assumptions, the number of fetal blood flow paths through the Nf^th^ bifurcation level becomes 2^Nf^. Therefore, the convective resistance in each channel in bifurcation Nf was defined as $\;R_{\mathrm{conv}.}^{b}(\text{Nf})=2^{\text{Nf}}R_{\mathrm{conv}.}^{b}(0)=2^{\text{Nf}}(128\mu_{b}L_{0}/\pi d_{0}^{4})$. In addition, the symmetrical model geometry resulted in an equivalent pressure across all channels at a given bifurcation level. Thus, one may consider that the channels operated in parallel and add all the parallel resistors together. In conclusion, the total resistance in a bifurcation Nf can be given by $R_{\mathrm{conv}.}^{b}(\text{Nf})=2^{k}R_{\mathrm{conv}.}^{b}(0)\text{/}2^{k}=128\mu_{b}L_{0}\text{/}\pi d_{0}^{4}$. Ultimately, because the bifurcations act in a series, the overall convective resistance of the feto-placental vasculature tree with umbilical artery at Nf = 0 plus *N* − 1 bifurcation level can be obtained as

1.3 $$R_{\mathrm{conv}.}^{b}(N)=\overset{N-1}{\underset{k=0}{\sum }}R_{\mathrm{conv}.}^{b}(k)=(N)R_{\mathrm{conv}.}^{b}(0)=\frac{128\mu_{b}L_{0}}{\pi d_{0}^{4}}(N).$$

Therefore, this equation expresses that the total convective resistance through the channels is the summation of all the channels' resistances by considering 2^Nf^ number of fetal blood flow paths (channels). It should be noted that each bifurcation added an additional convective resistance to the system, which had to be considered in the analytical model. We calculated the bifurcation resistance in the system by defining the energy dissipation and the pressure drop occurring between upstream and downstream of the bifurcation. We defined the overall bifurcation resistance of a structure with the umbilical artery at Nf plus *N* − 1 bifurcation levels as [29] $R_{\mathrm{bifur}.}^{b}(N){|}_{\mathrm{total}}={\dot{m}}_{0}(1-2^{-2N/3})\text{/}758\rho_{b}L_{0}\mu_{b}$. By describing the value of ${\dot{m}}_{0}$ = 8.33 × 10^−6^ kg s^−1^ (i.e. the fetal blood mass flow rate at the umbilical artery) and using values available in the literature [14,23,50] such as *L*_0 _≈ 0.3–0.7 m, the viscosity and density of blood [14] 1.7 × 10^−9^ Pa s and 10^3^ kg m^−3^, respectively, the bifurcation resistance in the human placenta becomes 1.82 × 10^−8^(1–2^−2*N*/3^) kg s^−1^. For the mouse placenta, the bifurcation resistance was 5.16 × 10^−7^(1–2^−2*N*/3^) kg s^−1^, where ${\dot{m}}_{0}$ = 8.76 × 10^−6^ kg s^−1^ as mentioned by [51], *d*_0_ = 0.54 mm [36] and *L*_0_ = 5.6 mm [45]. These bifurcation resistances can be neglected because the values are very small in relation to channel resistances.

#### 1.1.2. Diffusive resistance through the terminal villi

Mass diffusion occurs between the fetal and maternal blood through the trophoblast cells separating fetal capillaries and maternal blood. While oxygen diffuses from maternal blood across the trophoblast membrane and into the fetal haemoglobin, carbon dioxide diffuses in the opposite direction. Therefore, the total oxygen current for the fetal capillary can be given by

1.4 $${\dot{m}}_{\mathrm{ox}}=2^{N}\frac{D_{\mathrm{ox}}A\Delta n}{\delta }.$$

Here we assume the feto-placental vasculature system ends with 2^*N*^ number of terminal villi. Also, *δ* is the thickness of the placental barrier separating fetal and maternal blood, as shown in figure 2*b*, and is assumed to be roughly 0.2 *d*_fetal capillary_ [52]. In addition, $\;{\dot{m}}_{\mathrm{ox}}$ (kg s^−1^) is the gas mass flow rate, *A* is the surface area where the gas exchange occurs, *Δn* is the difference between gas concentration at the entrance of the fetal capillary and its surface. For simplicity, we also used ft as an abbreviation for fetal capillary in the equations. Taking into account that $\Delta n=C_{\mathrm{ox}}{|}_{\mathrm{ftcapillary}}\Delta P\text{/}\rho_{b}R_{\mathrm{ox}}^{\prime }T$ and assuming that the chemical potential of oxygen does not vary over the fetal capillary surface, one can simplify equation (1.4) as

1.5 $${\dot{m}}_{\mathrm{ox}}=2^{N}\frac{{D_{\mathrm{ox}}C_{\mathrm{ox}}|}_{\mathrm{ftcapillary}}\Delta PA}{\rho_{b}{R^{\prime }}_{\mathrm{ox}}T\delta }.$$

Here, *D*_ox_ is the diffusion coefficient of oxygen (because we are focusing on the oxygen transport), *T* is the temperature, $R_{\mathrm{ox}}^{\prime }$ is the gas constant for oxygen and *C*_ox_|_ft capillary_ is the concentration of oxygen in the fetal capillary, and Δ*P* is the partial pressure of oxygen in blood. Recent advances in capillary modelling with respect to oxygen transfer suggest that fetal vessels have a complex geometry [16,19,20]. In this study, using a lumped model to describe a network of fetal capillaries with a single effective length and diameter we assume the fetal capillary has a cylindrical shape with diameter *d*_ft_ and length *h*_ft_ where *d*_ft_ = *h*_ft_ (figure 2*b*). Thus, the total area of the fetal capillary can be expressed as *A* = 3π *d*_ft_^2^/2. By considering the fact that oxygen diffuses at 2^*N*^ fetal capillaries and *β* is the number of terminal villi and each terminal villous has one capillary (i.e. the terminal branch of a villous tree), the overall mass of oxygen diffusing to the fetal capillary can be obtained as

$${\dot{m}}_{\mathrm{ox}}=2^{N}\beta \frac{{D_{\mathrm{ox}}C_{\mathrm{ox}}|}_{\mathrm{ftcapillary}}\Delta P(3\pi \text{/}2){d_{\mathrm{ft}}}^{2}}{\rho_{b}{R^{\prime }}_{\mathrm{ox}}T(0.2d_{\mathrm{ft}})}.$$

In this study, the number of terminal villi *β* was assumed to vary from 60 to 70 in the human placenta. We also assumed the same value in the mouse placenta. Then, the total diffusive resistance through the system can be obtained as

1.6 $$R_{\mathrm{diff}.}(N)=\frac{\Delta P}{Q_{\mathrm{ox}}}=\frac{\rho_{b}{R^{\prime }}_{\mathrm{ox}}T(0.2)\rho_{\mathrm{ox}}}{2^{N}\beta {D_{\mathrm{ox}}C_{\mathrm{ox}}|}_{\mathrm{ftcapillary}}3\pi \text{/}2d_{\mathrm{ft}}}.$$

Next, we define the diameter of the fetal capillary *d*_ft_. Because the measurement of *d*_ft_ is challenging and requires a detailed analysis of the vasculature system in order to define *d*_ft_, we utilize the modelling assumption described by [28] to derive the optimal number of bifurcation levels that lead to the easiest fetal blood flow, as well as oxygen transfer through the vasculature system. This diameter has been determined as the difference between the overall lengths *L* of the feto-placental vasculature tree with infinite bifurcations and that of an actual tree with *N* bifurcation levels. By considering the flow through the fetal capillaries as a laminar flow, as discussed in previous section, one can consider the values of the two dimensionless ratios, as $d_{N}\text{/}d_{N-1}=2^{-1/3}$ and $L_{N}\text{/}L_{N-1}=2^{-1/3}.$ We then express *d*_ft_ as $d_{\mathrm{ft}}+\Sigma_{k=0}^{N}(L_{k})=\Sigma_{k=0}^{\infty }(L_{k})$; therefore, *d*_ft_ can be simplified as $d_{\mathrm{ft}}=4.85L_{0}\text{/}2^{(N+1)/3}$, where *L_N_* is the length of a tree with *N* generations and *L*_0_ stands for the length of the umbilical artery. Hence, the total diffusion resistance in the terminal villi can be obtained as

1.7 $$R_{\mathrm{diff}.}(N)=\frac{0.00875\rho_{b}{R^{\prime }}_{\mathrm{ox}}T\rho_{\mathrm{ox}}2^{(-2N+1)/3}}{{\beta D_{\mathrm{ox}}C_{\mathrm{ox}}|}_{\mathrm{ftcapillary}}L_{0}}.$$

Consequently, taking into account that ${\dot{m}}_{\mathrm{ox}}=Q_{b}[C_{\mathrm{ox}}{|}_{\mathrm{umbilical}\;\mathrm{artery}}-C_{\mathrm{ox}}{|}_{\mathrm{ftcapillary}}]$ the resistance of the *N*^th^ generation, that is the summation of the convective resistance of the last 2^*N*^ terminal villi plus the diffusive resistance of the terminal villi, can be given by

1.8 $$R(N){|}_{\mathrm{total}}=\frac{128\mu_{b}\rho_{b}L_{0}}{\pi d_{0}^{4}[C_{\mathrm{ox}}{|}_{\mathrm{umbilical}\;\mathrm{artery}}-C_{\mathrm{ox}}{|}_{\mathrm{ftcapillary}}]}+\frac{0.00875\rho_{b}R_{\mathrm{ox}}^{\prime }T\rho_{\mathrm{ox}}2^{(-2N+1)/3}}{\beta D_{\mathrm{ox}}C_{\mathrm{ox}}{|}_{\mathrm{ftcapillary}}L_{0}}.$$

Finally, the total resistance to the oxygen through the vasculature system for both human and mouse placentas can be expressed as

1.9 $$\begin{array}{rl} R(N){|}_{\mathrm{total}} & =R_{\mathrm{conv}.}(N)+R_{\mathrm{diff}.}(N)\\ & =\frac{1}{\alpha }\left[\frac{128\mu_{b}\rho_{b}L_{0}(N+1)}{\pi d_{o}^{4}[C_{\mathrm{ox}}{|}_{\mathrm{umbilical}\;\mathrm{artery}}-C_{\mathrm{ox}}{|}_{\mathrm{ftcapillary}}]}+\frac{0.00875\rho_{b}R_{\mathrm{ox}}^{\prime }T\rho_{\mathrm{ox}}2^{(-2N+1)/3}}{\beta D_{\mathrm{ox}}C_{\mathrm{ox}}{|}_{\mathrm{ftcapillary}}L_{0}}\right]. \end{array}$$

This equation describes an expression of the total resistance through the feto-placental vasculature system by considering the combination of convective and diffusive resistance. Where *α* takes into account the number of cotyledons, which equals a value between 60 and 100 in the human placenta [39,40]. In this study, the value of *α* has been considered to be 80 and one for human and mouse placenta, respectively. In addition, we assumed all cotyledons in both human and mouse placenta are actively used to transport the mass between fetal and maternal blood flow. To determine mathematically the optimal fetal blood flow through the fetal vasculature trees, equation (1.9) had to be minimized. It should be noted that *C*_ox_|_umbilical artery_ and *C*_ox_|_ft capillary_ are the concentrations of oxygen in fetal blood and are a function of the partial pressure of oxygen, which will be computed in the following section.

## 2. Concentration of oxygen dissolved in the fetal blood plasma and bound to haemoglobin

The concentration of oxygen bound to haemoglobin is related to the partial pressure of oxygen in the plasma *P*o_2_, and can be derived using Hill's equation as

2.1 $$\begin{array}{rl} C_{\mathrm{bound}\;\mathrm{ox}} & =C_{\max}S(P{\text{o}}_{2}),\\ S(P{\text{o}}_{2}) & \equiv \frac{{\left(K_{\mathrm{Hill}}P{\text{o}}_{2}\right)}^{\alpha }}{1+{\left(K_{\mathrm{Hill}}P{\text{o}}_{2}\right)}^{\alpha }}. \end{array}\}$$

Here, *C*_bound_ _ox_ is the concentration of oxygen bound to haemoglobin and *C*_max_ is the oxygen content of fetal blood at 100% haemoglobin saturation. By considering *C*_max_ for fetal blood, *C*_max_ = 9.82 mol m^−3^ as reported by [16]. Additionally, *K*_Hill _≈ 0.04 mm Hg^−1^ and *α* ≈ 2.65 are coefficients of Hill's equation [53], one can find the concentration of oxygen bound to haemoglobin in the fetal side.

The partial pressures of the umbilical artery and umbilical vein were found in prior studies [54,55] to be *P*o_2_ = *P*_ox_ = 15.7 mmHg at the umbilical cord and *P*_ox_ = 28.3 mmHg at the fetal capillary. Therefore, using Hill's equation, one can compute *C*_bound ox_ = 1.647 mol m^−3^ and *C*_bound ox_ = 4.244 mol m^−3^ in the umbilical artery and umbilical vein, respectively. Note that we have assumed that the value for the umbilical vein is the same as the value in the fetal capillary.

In addition, using Henry's law, we can define the dissolved oxygen concentration in the fetal blood plasma *C*_diss ox_ ≈ 0.13 mol m^−3^, which corresponds to an oxygen content of 3 ml oxygen/l blood or a partial pressure of 13 kPa under normal conditions [16,56]. Therefore, we define the total concentration of oxygen in blood by adding the concentration of oxygen bound to haemoglobin and oxygen dissolved in the fetal blood plasma as *C*_ox_|_umbilical artery_ = 1.777 mol m^−3^ and *C*_ox_|_ft capillary_ = 4.374 mol m^−3^ for human placenta. We also assumed the same values for the mouse placenta. Finally, these values were substituted in equation (1.9) to examine the total resistance to oxygen transport through the vasculature system for both human and mouse placentas.

## 3. Results and discussion

As can be seen in equation (1.9), the convective part of the resistance increases linearly with the number of bifurcations, *N*. However, the diffusive part decreases as *N* increases. To achieve the minimum mass transfer resistance, $R^{\text{ox}}{\left(N\right)}_{\mathrm{total}}$ the number *N* must lead to the most effective mass transfer from the fetal blood to the maternal blood. To determine the optimal value of *N*, we took the derivative of equation (1.9) with respect to *N* and set it to zero. We then focused on the optimization of both human and mouse placental vasculature systems as follows.

### 3.1. Optimization of the human feto-placental vasculature system

**Figure 3.**
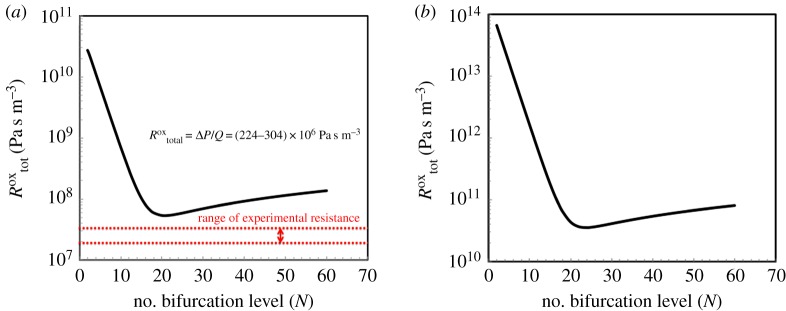
Optimum number of bifurcation level *N*. (*a*) Human and (*b*) mouse. The minimum resistance to oxygen access corresponds to *N* = 18 where $\log[R_{\mathrm{total}}^{\mathrm{ox}}(N)]=7.77$ for human placenta and *N* = 22 where $\log[R_{\mathrm{total}}^{\mathrm{ox}}(N)]=10.55$ for mouse placenta.

Some values for the parameters reported in this equation are listed in table 1 for human and mouse. Oxygen properties were taken at 36°C, namely *R′*_ox_ = 259.8 J kg^−1^ K^−1^ and *ρ*_ox_ = 0.0468 kg m^−3^ at the fetal capillary. We have also considered the average umbilical artery length as 0.5 m. After substituting experimentally measured values into equation (1.9), the optimum level of bifurcation *N* for the human placenta yields 18. Figure 3*a* shows the logarithmic form of the total resistance to oxygen between the entrance of the umbilical arteries and the feto-placental capillaries as a function of the level of bifurcation *N* in the human placenta. As can be seen from figure 3*a*, the minimum value of total resistance is achieved when *N* = 18. For comparison, we have also calculated the total resistance to oxygen using the experimental reported values [21] and drawn the variation of $R_{\mathrm{total}}^{\text{ox}}$ in the figure. The discrepancy between the predicted optimal value of our model and the measured value of $R_{\mathrm{total}}^{\text{ox}}$ might be because following other researchers [21], we also assumed the experimental parameters of Δ*P*. Thus, the exact measured values of Δ*P* would significantly reduce this discrepancy. Replacing the value of *N* in equation (1.9), the total resistance of the human placenta ($R_{\mathrm{total}}^{\text{ox}}(N)$) can be calculated as 5.95 × 10^7 ^Pa s m^−3^. Therefore, an optimal fetal blood flow resistance is $\log[R_{\mathrm{total}}^{\text{ox}}(N)]=7.77$. Consequently, by having the number of bifurcation level *N* and using the reported values of the fetal blood properties in table 1, one can calculate the optimal total length of the feto-placental vasculature system as *L*_tot_ = *L*_0_ + *L*_1_ + … + *L*_N_ = *L*_0_ + (2^−1/3^*L*_0_) + (2^−1/3^)^2^*L*_0_ + … + (2^−1/3^)^N^*L*_0_ = 2.34 m. Using this idealized model, we have also defined the optimum volume flow rate of oxygen through the fetal capillary as ${\dot{m}}_{\mathrm{ox}}$/*ρ*_b_ = *πd*_0_^4^Δ*P*_tot._/128*μ*_b_*L*_0_(*N* + 1) = 25.13 ml min^−1^.

The number of bifurcation level *N* predicted using our model is consistent with the values reported in several studies. For instance, researchers have stated that, from the stem villi, there are ‘up to four’ branching generations and then ‘two to 30 (mean 10) more generations of unequal dichotomous branching’ [34]. They suggested on average 14 generations of asymmetric villus branching. Others have reported a value of around 11 generations of villous tree and 4–5 generations in the chorionic vessels [23,37]. In addition, the parametrizations of models of the feto-placental vasculature proposed by [57] suggested 15 branching generations. The predicted number of bifurcation level *N* of the fetal capillary agrees with the measured value, which affirms that our model's estimate is reasonable. We have also estimated the ‘optimal’ fetal capillary diameter with respect to the oxygen transport, by applying the values reported in table 1 and equation (1.6), as *d*_ft_ = 300 µm. The diameter of the fetal capillary of the vasculature system has been measured and reported as ranging from 30 to 80 µm [34,58–60]. The discrepancy between the predicted optimal value and the measured value of fetal capillary diameter could be an effect of our idealized model and of the assumptions used to derive the model, as will be discussed in detail in the next section.

### 3.2. Optimization of the mouse feto-placental vasculature system

The mouse placenta was modelled in a similar manner. We used the literature values listed in table 1 to model the vasculature system in the mouse strain CD-1 at embryonic days (E) 17.5. Using these parameters, *N* is calculated as 22. The total resistance of the mouse placenta $R_{\mathrm{total}}^{\text{ox}}(N)$, was calculated by plugging *N* into equation (1.9) as 3.56 × 10^10^ Pa s m^−3^. Thus, an optimal fetal blood flow resistance can be calculated as $\log[R_{\mathrm{total}}^{\mathrm{ox}}(N)]=10.55$. Figure 3*b* shows the total resistance of the mouse placenta versus the level of bifurcation *N*. As can be observed from figure 3*b*, the minimum value of total resistance corresponds to *N* = 22. By setting the number of bifurcation level *N* at embryonic days (E)17.5 and taking into account the minimum/maximum reported values of the fetal blood properties in table 1, our model predicts that the total optimum volume flow rate of oxygen is ${\dot{m}}_{\mathrm{ox}}$/*ρ*_b_ = π*d*_0_^4^Δ*P*_tot._/128*μ*_b_*L*_0_(*N* + 1) = 0.135 ml min^−1^, and the ‘optimal’ diameter of the fetal capillary using equation (1.6) is *d*_ft_ = 28 µm. We also defined the optimal total length of the feto-placental vasculature system computed as *L*_tot_ = *L*_0_ + *L*_1_ + … + *L*_N_ = *L*_0_ + (2^−1/3^*L*_0_) + (2^−1/3^)^2^*L*_0_ + … + (2^−1/3^)^N^*L*_0_ = 26.56 mm for mouse placenta.

There are 23.7 ± 1.3 generations of fetal villi in the mouse placenta as experimentally reported [61], which is consistent with the predicted value of *N* based on our proposed model. In addition, the mean diameter of the fetal capillary has been reported as 10.5 µm at embryonic days (E)18.5 [62]. Thus, *N* agree well with the predicted values using our model.

To compare the model-predicted results with the biological data in more detail, we used the values reported previously in the literature [33,62] for the mouse placenta. In general, due to the *in vivo* limitations and the complicated manipulation of *ex vivo* organs, experimental analyses of feto-placental vasculature are very challenging. In addition, two-dimensional histological sections are not reliable due to the complexity of the geometry. In the reported experiments, 3–16 casts of the feto- and materno-placenta vasculature system were examined from several pregnant mice at different gestational ages from E12.5 to term. The mean diameters of the fetal capillary for different gestational ages are shown in table 2, and those values showed close agreement with the predicted theoretical model. Our optimum fetal capillary diameters are roughly two times higher than the experiments, but the experimental data also largely vary as is shown in table 1. Note that this model is an idealized model and we obtained the optimized diameter; thus, one might expect the values defined theoretically to be different from the experimental values. In addition, individual variations in branching patterns could have a large effect on this model, which needs to be taken into account when analysing the results. It should also be noted that some experimental values were derived from measurements made on corrosion casts after birth under visual examination, which made variations in branching patterns difficult to detect. This might have caused experimental errors.

**Table 2.** Comparison between experimental measurements of 3 to 16 casts of feto-placental vasculature systems and analytical predictions of the fetal capillary for mouse placenta at different stages of gestation.

stages of gestation	E12.5	E14.5	E16.5	E18.5	ref.
mean diameter of fetal capillary (µm) in the mouse placenta	14.07 ± 1.36	14.57 ± 0.37	11.77 ± 0.21	10.47 ± 0.43	[62]
	15	15	15	15	[33]
	28	28	28	28	the model proposed by this study

**Figure 4.**
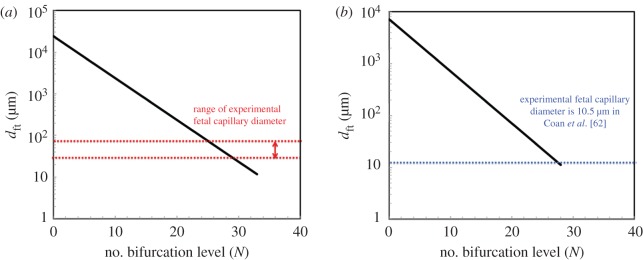
Variation of the diameter of the feto-placenta vasculature *d*_ft_ versus bifurcation level *N*. (*a*) Human and (*b*) mouse placenta. All other parameters have been reported in table 1.

We further analysed the variation of the diameter of the fetal capillary versus bifurcation level *N*, calculated based on the analytical model, for both human and mouse placenta and compared them with the experimental measurements as shown in figure 4*a,b*. As can be seen in figure 4*a*, the measured *d*_ft_ varies from 30 to 80 µm [34,58–60] where the predicted value of *d*_ft_ for bifurcation level 18 calculated as 300 µm. Figure 4*b* also shows that the measured average diameter of the fetal capillary is 15 µm [33,62] where the value of *d*_ft_ for 22 bifurcation level calculated as 28 µm. We can conclude that the predicted diameter of the fetal capillary is close to the measured values, thus our model's estimate appears reasonable. The discrepancy between the data could be due to the variation of experimental measurements as well as the assumptions made in the modelling analysis as follows.

It should be emphasized that some parameters are critical in order to decrease the discrepancy between the experimental data and predicted results at each step to define equation (1.9) including: (1) the diameter and the length of the fetal capillaries (i.e. *d*_ft_ and *h*_ft_) which play a critical role because they are both involved in the surface area where the gas exchange occurs, (2) the thickness of the placental barrier Δ**, separating fetal and maternal blood, (3) the length and diameter of the umbilical artery (i.e. *L*_0_ and *d*_0_), (4) *C*_ox_|_umbilical artery_ and *C*_ox_|_ft capillary_ that are the concentrations of oxygen in fetal blood and they are a function of the partial pressure of oxygen, (5) the partial pressure of umbilical artery and umbilical vein Δ*P*, (6) the number of cotyledons *α* and (7) the number of fetal capillaries *β*. All these parameters depend specifically on the experimental measurements. In the current study, they have been taken from the literature. However, these parameters impact the predicted resistance and the number of bifurcation level *N* in the vasculature system. Therefore, careful measurements of these data could decrease the discrepancy between the analytical predictions and experimental data.

### 3.3. Model limitations

We have made several assumptions when constructing our analytical model: (1) the proposed model was created for oxygen transport only in the placenta; (2) the diffusion within the fetal vasculature tree, where oxygen is transported in the fetal blood, can be neglected and diffusion is the main method of oxygen transport in the fetal capillaries; (3) the bifurcation level from the umbilical artery to the fetal capillary was assumed to be symmetrical where the structure of the vessels is asymmetric and highly heterogeneous [23,24]; (4) the length of the fetal capillary was equal to its diameter; (5) similar bifurcations occurred throughout the tree, even though the bifurcation levels in different branches were not equal *in vivo* [39,41]; (6) there was negligible pulsatile fetal blood flow in the umbilical cord; (7) assumptions were made to determine the diameter and the length of the vasculature system; (8) all the fetal capillaries have a cylindrical shape, where recent advances suggest that fetal vessels have a complex geometry [16,19]; (9) all species in the system have the same chemical potential; (10) the model is two dimensional and it ignores the arterioles and venules as well as constant trophoblastic membrane thickness among others; (11) although the placental villi from their arterial origin to the lacunae are morphologically separated into conductive, intermediate, and terminal villi, herein, we considered only conductive and terminal villi; (12) by assuming the oxygenation value for umbilical vein is identical to feto-placental capillary we neglected the whole villous venous tree and placental metabolism that could have a profound effect on oxygen transfer; (13) we neglect all the convective resistances in the capillaries; and (14) we assumed the number of terminal villi *β* to vary from 60 to 70. Some variables not considered in this model include: (1) the diffusion of nutrients and waste, (2) variabilities in fetus size and weight, (3) variability in maternal blood saturation and the partial pressure of oxygen based on the oxygen demands of the placenta, and (4) the number of cotyledons and fetal vessel capillaries in both human and mouse placentas. Additionally, in the proposed model, the convective resistance was defined by the ratio between the pressure drop of the fetus heart and the effects of feto-placental capillaries on the blood flow rate. However, the heart rate could increase to compensate for reduced oxygen supply, which is a transient response and may not be a linear process. These are subjects for potential future investigation.

Despite these limitations, (1) this proposed ‘mixed model’ is the first of its kind to provide a detailed analysis of a geometrical structure of the placenta in which both ‘conductive’ and ‘terminal’ villi are considered at the end of single (in human) or multiple (in mouse) pregnancies, (2) the proposed idealized model is straightforward and adaptable which enables using the parameters that are easy to measure or available in engineering/biological resources, (3) using this model, one can define some of the parameters such as the values of oxygen volume flow rate through the vasculature system, the diameter of fetal capillaries and the length of feto-placental vasculature that are usually hard to measure experimentally, (4) the proposed model is a first step to understand and model the detailed structure and oxygen diffusion across the trophoblast membrane between the fetal and maternal red blood cells in the feto-placental vasculature system in both human and mouse placentas, and (5) because the model is based on the geometry of the placenta vasculature tree as well as the properties of mass exchange, such as concentration, it could be used to understand how other substances, such as nutrients and viruses, transfer between the mother and fetus. It is expected that future works would intricate this model, by adding more detail, such that it can be used for a specific application or a clinical condition.

## 4. Conclusion

In this study, we formulated a quasi-steady mass transfer model for oxygen exchange between fetal and maternal blood in mice and humans. We proposed a ‘mixed model’ whereby both ‘conductive’ and ‘terminal’ villi are presumed to be present at the end of single (in human) or multiple (in mouse) pregnancies. Summing up both parts of the villous tree, and assuming that the matching occurs at the 15 generation, we predict an optimal number of 18 and 22 bifurcation levels in the human and the mouse placentas, respectively. We then used the resulting calculations to define the dimensions of the feto-placental vasculature, such as total length, the optimum diameter of the fetal capillary and the oxygen volume flow rate through the system. Although these parameters are difficult, time consuming and costly to measure experimentally, wherever possible, we compared our model predictions with experimental data reported in the physiology literature [34,36,51,58,59,62,63] related to human and mouse placentas. It was shown that the number of bifurcation levels *N* agreed closely with the reported experimental results in both human and mouse placentas. Wherever possible, we also compared the predicted optimal diameter of the terminal villi in the mouse placenta with experimentally measured data.

In particular, the focus of this research was to create an ideal architecture of both human and mouse placental structures, from umbilical artery to terminal villi, by applying a mathematical model of oxygen transport in the placenta. To obtain a reliable model that can be appropriate for the actual placenta, the geometry and the oxygen transport problem should adhere as closely as possible to the *in vivo* state. Several assumptions were made when creating the model including the two-dimensional structure of the placenta, the consideration for only oxygen transport in the placenta, the symmetrical structure of the vasculature system, to name a few. This study presents the first attempt to represent the complex structure of the feto-placental vasculature system from the large to small vessels that contribute to the mass exchange between the mother and her fetus. In addition, because the analytical model proposed in this study is based on the geometry of the placenta vasculature tree as well as the concentration of species, it can be used as a stepping-stone to understand how other substances, such as nutrients and viruses, transfer through the placenta.

This research also holds promise for biologists and engineers. It offers an example for biologists interested in analysing the optimal number of bifurcation level *N* with respect to oxygen transfer in both human and mouse placentas. It also presents a valuable insight to engineers who hope to design advanced placental treatment devices. This study also has the potential to serve as a first step towards developing new concepts for designing artificial feto-placental vasculature systems to better understand how the feto-placental vasculature system functions.

## Data accessibility

The data are deposited at Dryad Digital Repository: https://dx.doi.org/10.5061/dryad.j8h41. [64]
